# Achieving 0.05 Ω-mm contact resistance in non-alloyed Ti/Au ohmics to *β*-Ga_2_O_3_ by removing surface carbon

**DOI:** 10.1063/5.0276786

**Published:** 2025-06

**Authors:** Naomi Pieczulewski, Kathleen T. Smith, Corey M. Efaw, Arjan Singh, Cameron A. Gorsak, Joshua T. Buontempo, Jesse Wensel, Kathy Azizie, Katie Gann, Michael O. Thompson, Darrell G. Schlom, Farhan Rana, Hari P. Nair, Steven M. Hues, Elton Graugnard, Paul H. Davis, Debdeep Jena, Huili Grace Xing, David A. Muller

**Affiliations:** 1 Department of Materials Science and Engineering, Cornell University, Ithaca, New York 14853, USA; 2 School of Applied and Engineering Physics, Cornell University, Ithaca, New York 14853, USA; 3 Micron School of Materials Science and Engineering, Boise State University, Boise, Idaho 83725, USA; 4 School of Electrical and Computer Engineering, Cornell University, Ithaca, New York 14853, USA; 5 Micron Technology, 8000 S Federal Way, Boise, Idaho 83707, USA; 6 Kavli Institute at Cornell for Nanoscale Physics, Cornell University, Ithaca, New York 14853, USA; 7 Leibniz-Institut fur Kristallzuchtung, Max-Born-Str. 2, Berlin 12489, Germany

## Abstract

Preserving a contamination-free metal–semiconductor interface in *β*-Ga_2_O_3_ is critical to achieve consistently low resistance (< 1 Ω-mm) ohmic contacts. Here, we report a scanning transmission electron microscopy study on the variation in Ti/Au ohmic contact quality to (010) *β*-Ga_2_O_3_ in a conventional lift-off vs a metal-first process. We observe a thin ∼1 nm carbon barrier between the Ti and Ga_2_O_3_ in a non-conductive contact fabricated by a conventional lift-off process, which we attribute to photoresist residue, not previously detected by x-ray photoelectron spectroscopy due to the thinness and patchy coverage of the carbon layer, as well as roughness of the Ga_2_O_3_ surface. This thin carbon barrier is confirmed by electron energy loss spectroscopy and atomic force microscopy-infrared spectroscopy. We believe that the presence of the thin and patchy carbon layer leads to the highly inconsistent contact behavior in previous reports on non-alloyed contacts. Adventitious carbon is also observed in a conductive ohmic contact metal-first processing on an as-grown sample. We find that a five minute active oxygen descum is sufficient to remove this carbon on as-grown samples, further improving the ohmic behavior and reducing the contact resistance R_c_ to 0.06 Ω-mm. We also show that an hour long UV-ozone treatment of the Ga_2_O_3_ surface can eliminate carbon residue from the lift-off processing, resulting in a low R_c_ of 0.05 Ω-mm.

## I. INTRODUCTION

Since the first demonstration in 2012, *β*-Ga_2_O_3_ transistors have been extensively investigated toward commercialization.^1^ Although Si currently dominates the transistor market, higher bandgap semiconductors offer superior critical electric field tolerance for more efficient high-power devices. SiC and GaN were the first wide-bandgap semiconductors to achieve commercial success. *β*-Ga_2_O_3_’s bandgap (4.8 eV) is greater than those of both SiC (3.3 eV) and GaN (3.4 eV), setting a higher theoretical limit to the Baliga figure of merit, which describes the fundamental trade-off in power electronics between breakdown voltage and device on resistance.^2,3^ *β*-Ga_2_O_3_ is also an economically viable and commercially scalable technology, due to the availability of melt-grown native substrates.^4,5^ Doping densities up to 5 × 10^20^ cm^−3^ have been demonstrated through various intrinsic and extrinsic doping methods for both channel and selective-area contact regions.^3,6–11^

Despite fundamental demonstrations of lateral and vertical *β*-Ga_2_O_3_ devices with high breakdown voltages and critical electric fields, reaching the material’s theoretical limit remains a challenge.^12–16^ Among the many key challenges to overcome, high resistance at the source/drain ohmic contacts persists as a key obstacle. The difficulty partially arises from the lack of suitable low-work function metals for ultra-wide bandgap semiconductors with large electron affinity. Many studies have focused on using a Ti low work function contact layer with an Au overlayer; however, linear ohmic contacts were obtained only after annealing at temperatures near 400–450 °C,^17–19^ except in the studies by us (Smith *et al.*^20,21^). Ti has been found to scavenge oxygen from the *β*-Ga_2_O_3_ surface forming a Ti–TiO_*x*_ layer at the interface. This intermediate layer partially matches the lattice to defective Ga_2_O_3_ and facilitates charge transport.^22,23^

Smith *et al.* also reported extreme variability in performance of non-alloyed Ti contacts formed by lift-off, with current differing by twelve orders of magnitude between devices fabricated by identical methods and by six orders within a single die.^20^ The inconsistency highlights severe process variation in the conventional contact lithography process and a need for deeper understanding of the Ti–Ga_2_O_3_ interface. In our previous work,^20^ we demonstrated consistent, linear ohmic IV characteristics using non-annealed Ti/Au contacts processed with a metal-first method on heavily doped Ga_2_O_3_. This Ti/Au stack forms a Schottky contact that, despite having an energy barrier, can exhibit linear ohmic IV behavior via thermionic field emission in degenerately doped Ga_2_O_3_. These studies confirmed that other low work function metals, such as Al and Cr, form similar Schottky barriers and also exhibit linear ohmic behavior to degenerately doped material.^21^ Moreover, we found that a pristine Ga_2_O_3_ surface is critical for successful ohmic contact formation.^20^

In this work, we correlate the ohmic behavior of non-annealed Ti/Au contacts on heavily doped (010) *β*-Ga_2_O_3_ with its interface structure and chemistry. Scanning transmission electron microscopy (STEM), atomic force microscopy-infrared spectroscopy (AFM-IR), and time-of-flight secondary ion mass spectra (ToF-SIMS) measurements reveal that carbon contamination can persist after the removal of photoresist patterns, inhibiting electrical conduction in the conventional lift-off fabrication process. We further show that ambient carbon contamination can be measured by electron energy loss spectroscopy (EELS) and electron spin resonance (ESR) spectroscopy and increases the contact resistance in a metal-first process that does not use photoresist. We demonstrate that sufficient cleaning of the Ga_2_O_3_ surface by UV-ozone or oxygen plasma descum achieves low-resistance contacts.

## II. METHODS

In this study, the contact electrical properties for a total of six samples are compared: sample A, B, B′, B″, C, and D. Five of the *β*-Ga_2_O_3_ films (all except sample B″) were grown by metal organic chemical vapor deposition (MOCVD) on Fe-doped (010) *β*-Ga_2_O_3_ substrates in an Agnitron Agilis 100 system. Before loading into the reactor, the samples were dipped in a 48% HF bath for 30 min to reduce interfacial Si.^24,25^ Detailed growth conditions, layer thicknesses, their measured carrier concentration, sheet resistance (*R*_sh_), and mobility can be found in the supplementary material section. The MOCVD grown samples were diced and solvent cleaned after growth. The sixth sample B″ used suboxide molecular beam epitaxy (*S*-MBE) to grow a 1 *μ*m thick Si-doped *β*-Ga_2_O_3_ film with a carrier concentration of 3.0 × 10^19^ cm^−3^, confirmed by secondary ion mass spectroscopy (SIMS), and a mobility of 62 cm^2^/V-s, confirmed by Hall measurements.^20^

Samples A, B′, B″, C, and D were *in situ* doped, while sample B was *ex*-*situ* doped by ion implantation. Details of ion implantation conditions can be found in the supplementary material section, and the annealing conditions are given in Ref. 7. All relevant sample information is summarized in Table I along with samples used for ESR, AFM-IR, and ToF-SIMS characterizations.

**TABLE I.** Summary of *β*-Ga_2_O_3_ samples, non-alloyed Ti/Au contact processes, and resultant specific contact resistances.

Sample	Growth method	Doping method	*N*_d_ (cm^−3^)	*t*_epi_ (nm)	Contact process	*ρ*_c_ (Ω-cm^2^)	Characterization?
A^a^	MOCVD	*In situ*	9.8 × 10^19^	158.5	Mesa isolation^b^ + 2 min. O-descum	⋯	TEM
					+ lift-off + PR strip		
					+ acid strip + metal-first		
B	MOCVD	Ion Implantation	5.0 × 10^19^	150	Metal-first	1.2 × 10^−5^	TEM
B′	MOCVD	*In situ*	9.3 × 10^19^	215	Metal-first	3.2 × 10^−5^	No
B″^c^	*S*-MBE	*In situ*	3.0 × 10^19^	1000	Metal-first	1.4 × 10^−4^	No
C	MOCVD	*In situ*	9.0 × 10^19^	220	5 min. O-descum + metal-first	7.1 × 10^−7^	TEM
D	MOCVD	*In situ*	9.4 × 10^19^	215	UV-ozone + lift-off	4.2 × 10^−7^	TEM
E	MOCVD	UID	Insulating	550	⋯	⋯	ESR
F	MOCVD	UID	Insulating	550	30 min. HF + TBCl etch	⋯	ESR
G	Substrate	(010) Ga_2_O_3_:Fe	Insulating	⋯	PR expose + develop +2 min. O descum	⋯	AFM-IR & ToF-SIMS

^a^ Sample B in Smith *et al.*^20^

^b^ Contact area protected by hard mask but underwent photolithography process and possible heating of photoresist residue during ICP-RIE mesa etch.

^c^ Sample D in Smith *et al.*^20^

Transfer length method (TLM) patterns were fabricated on the samples to extract the contact resistance. For sample A, both linear and circular TLM (LTLM and CTLM) patterns were fabricated. The full process details can be found in Ref. 1. This sample first underwent mesa isolation by ICP-RIE and metallization by lift-off, but the contacts were found to be barely conducting. Subsequently, the metal contacts were removed, and the metal-first contact process was applied. However, the contacts were still found to be non-conducting, which made these samples particularly interesting for STEM examination. The complete fabrication process for sample A is summarized in the supplementary material. For samples B, B′, and B″, CTLM patterns were fabricated using the metal-first process identical to sample A. The Ti/Au contact layers were evaporated at a base pressure < 3 × 10^−8^ Torr. For sample C, the as-grown sample was treated with an oxygen active plasma descum for 5 min at 100 W. Ti/Au CTLM patterns were fabricated immediately afterward using the metal-first process. Finally, for sample D, CTLM patterns were photolithographically defined. The CTLM patterns were then treated in UV-ozone for an hour in a UVOCS T10X10/OES ultraviolet ozone cleaning system. The Ti/Au contact layers were then immediately deposited by electron-beam evaporation and then lifted off. The contact processes for all samples are summarized in Table I. Note that all contacts reported here are non-alloyed.

The CTLM patterns were measured using a Keithley 4200 semiconductor characterization system in a four-point probe configuration. The designed CTLM patterns have an inner radius of 50 *μ*m and a pad spacing of 5–12 *μ*m, and the dimensions confirmed by scanning electron microscopy are used in the TLM analysis.

Atomic scale characterization was performed using annular dark-field (ADF-STEM) imaging and EELS. Cross section lamellas of samples A–D were prepared using a Thermo Fisher Helios G4 UX focused ion beam. Protective C and Pt layers were deposited on the lamella and prepared with a final milling step of 2 keV to reduce damage. STEM measurements were taken with an aberration-corrected Thermo Fisher Spectra 300 CFEG. The ADF imaging was performed operating the microscope at 300 keV with a probe convergence semi-angle of 30 mrad. STEM–EELS was performed operating at 120 keV equipped with a Gatan Continuum spectrometer and camera with a spectral dispersion of 0.15 eV/ch. The inelastic background of each spectrum was removed by fitting and subtracting either a power law (for the Ti–L edge) or a decaying exponential function (for the C–K edge).

Field-modulated ESR spectroscopy was measured in a home-built spectrometer equipped with an X-band coplanar waveguide (CPW) resonator. ESR spectroscopy was performed with the magnetic field along the [010] axis of *β*-Ga_2_O_3_ at 10 K in a Lakeshore CPX-VF cryogenic probe station. The effect of ambient air exposure on the substrate surface was investigated through two samples of 550 nm unintentionally doped (UID) *β*-Ga_2_O_3_ MOCVD films grown on Fe-doped (010) *β*-Ga_2_O_3_ substrates. One of the substrates was only solvent cleaned prior to MOCVD growth (sample E), while the other was additionally treated in 48% HF for 30 min prior to loading into the reactor. The HF treated substrate, while in the reactor, was further subject to an *in situ* tert-butyl chloride (TBCl) etch at 750 °C^26^ prior to the start of the film growth (sample F). To ensure minimal contamination of the surface of the MOCVD film itself, both samples were transferred from the MOCVD reactor to the ESR spectrometer cryostat promptly after growth, spending less than ∼2 min in air. We note that the ESR measurement carried out in this work is designed to measure ESR signals in thin films several micrometers thick and requires insulating samples.

An Fe-doped (010) *β*-Ga_2_O_3_ substrate (sample G) was characterized by atomic force microscopy-infrared spectroscopy (AFM-IR) and time-of-flight secondary ion mass spectra (ToF-SIMS) to understand the contact surface. Sample G was patterned using the same photoresist spin-coating, exposure, and development conditions as the lift-off process used for samples A and D and subject to a subsequent two-minute 100 W active oxygen descum. A Bruker Anasys nanoIR3-s AFM-IR system equipped with an Angewandte Physik and Elektronik (APE) Carmina tunable pulsed broadband mid-infrared (IR) laser source was used for characterization to enable nanoscale near-field infrared spectroscopy chemical analysis and mapping. A pre-mounted gold-coated PR-EX-TnIR-D probe (Bruker) with a nominal tip radius of 20 nm was employed to reduce effects linked to IR absorption from the silicon-based cantilever.^27,28^ AFM-IR data were collected in tapping mode with the probe driven at its fundamental oscillation frequency (i.e., *f*_0_, ∼250 kHz) to image sample topography and the next higher mode (i.e., *f*_1_, ∼500 kHz) to detect IR absorption via photothermal expansion/contraction, with the Carmina’s pulse repetition rate phase-locked to the difference frequency (i.e., *f*_1_–*f*_0_) for resonance enhancement of the photothermal IR signal.

AFM-IR spectra and maps were acquired with Analysis Studio version 3.17. IR point spectra were measured over the range of 680–2040 cm^−1^ at a 1 cm^−1^ sampling interval with a fast Fourier transform (FFT) post-processing filter applied to smooth the data. AFM-IR maps were acquired in narrowband mode [20 cm^−1^ full width at half maximum (FWHM) spectral bandwidth] at a user-specified nominal IR wavenumber (1600 cm^−1^) corresponding to the absorption maximum of the most intense characteristic peak observed in the photoresist spectrum. Gwyddion version 2.63 was subsequently used for image processing of maps.^29^ Horizontal lapses in probe tracking of drastically rough features during imaging with the AFM probe were accounted for using a scar correction data processing module.

ToF-SIMS were acquired in both positive and secondary ion modes using a 25 keV ${Bi}_{1}^{+}$ primary ion beam. For the photoresist and open areas, region-of-interest spectra were obtained, where the detected signal was integrated over pixels only within specific areas of an ion image.

## III. RESULTS AND DISCUSSION

**FIG. 1.**
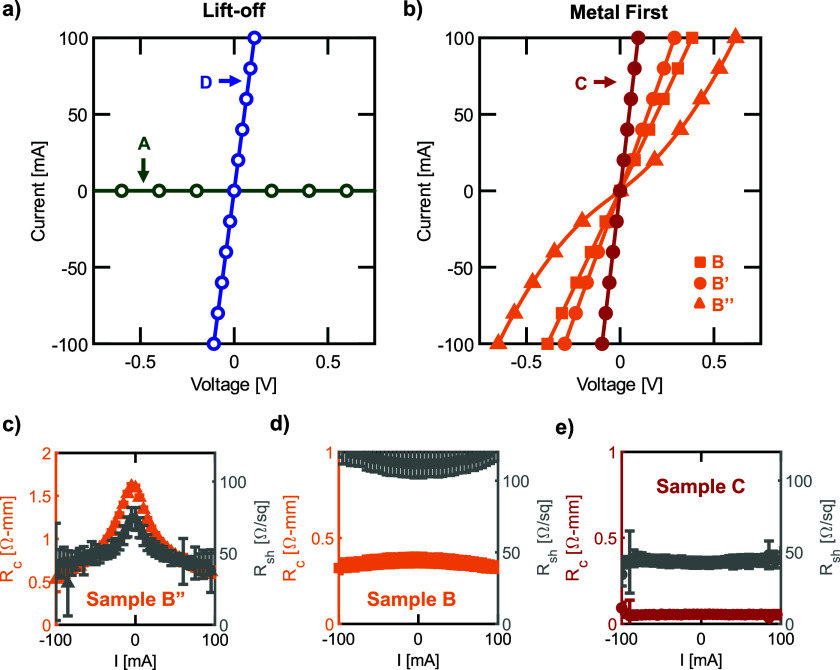
IV curves for a CTLM pattern with 5 *μ*m pad spacing for (a) lift-off processed sample A (green circles) and sample D (blue circles) and (b) metal-first processed sample B (orange squares), sample B′ (orange circles), sample B″ (orange triangles), and sample C (red circles). UV-ozone cleaning for lift-off sample D shows dramatic improvement in conductivity over sample A, which is non-conductive. The IV curves for metal-first samples B, B′, and B″ show that the growth method or doping method does not significantly affect the contact resistivity, but an oxygen plasma descum significantly improves the ohmic behavior seen in sample C. The linearity of the IV curves is further captured by the extracted *R*_c_ and R_sh_ data shown in (c)–(e). Linear contacts show no dependence in *R*_c_ and *R*_sh_ on applied current bias, while leaky Schottky contacts have lower *R*_c_ at higher applied bias as the reverse biased junction becomes dominated by tunneling current. The transition from leaky Schottky contact behavior for (c) sample B″ to linear contact behavior for (d) sample B and (e) sample C can be clearly seen in the *R*_c_ vs I curves. These curves for all samples are shown in Figs. S1–S5 in the supplementary material.

IV curves for a 5 *μ*m pad spacing are shown in Fig. 1. While the contacts fabricated by lift-off with a short active oxygen treatment on sample A (green) are non-conductive (such that *R*_c_ cannot be extracted), the addition of an hour long UV-ozone treatment prior to metal deposition (sample D, blue) led to contacts that are linear and ohmic [Fig. 1(a)]. All metal-first processed contacts shown in Fig. 1(b) are conductive and reasonably ohmic as deposited. Metal-first contacts fabricated on untreated ion-implanted (sample B, orange squares) and MOCVD-grown (sample B′, orange circles) degenerately doped samples are linear and ohmic. The contacts on the *S*-MBE-grown material (sample B″, orange triangles) are highly leaky Schottky due to the lower doping density (∼3 × 10^19^ cm^−3^). The metal-first contacts on MOCVD-grown material that received the active oxygen descum treatment (sample C, red circles) are also linear and ohmic with lower resistance [similar to sample D in Fig. 1(a)], as shown by the steeper slope of the IV curve. The linearity of the IV curves is also captured by the flat trends of the extracted contact resistance (*R*_c_) and sheet resistance (R_sh_) data shown in Figs. 1(c)–1(e) and the supplementary material.

The *R*_c_ and R_sh_ values are extracted using the CTLM method at an applied bias of 5 mA for samples B, B′, C, and D and a bias of 50 mA for sample B″ (due to the Schottky character of the contact that prevents accurate extraction of the contact resistance at low applied bias). All of the CTLM results are summarized in Table II. Error bars are calculated and reported for each CTLM pattern individually, using the standard deviation of the linear regression with a confidence interval of 95%. The range of measured values for R_c_, R_sh_, and specific contact resistance (*ρ*_c_) across the sample is also reported. For samples B, B′, and B″, measurements for which the uncertainty in either R_c_ or R_sh_ is >25% are considered unreliable and not included as part of the range. Samples C and D have contact resistances nearing the limit at which the CTLM patterns used here can give reliable measurements. As such, a more relaxed constraint is applied such that measurements for which the uncertainty in either R_c_ or R_sh_ is >50% are considered unreliable. Measurement certainty can be improved by incorporating CTLM patterns with more TLM pads and a greater range of pad spacing.

**TABLE II.** Summary of IV and range of TLM results on non-alloyed Ti/Au contact sample wafers.^a^

Sample	IV Behavior	*R*_c_ (Ω-mm)	*R*_sh_ (Ω/□)	*ρ*_c_ (Ω-cm^2^)	Bias (mA)
A	Non-conductive	⋯	⋯	⋯	⋯
B	Linear ohmic	0.37(4)–0.42(3)	95(7)–112(9)	1.2(2)–1.8(1) × 10^−5^	5
B′	Linear ohmic	0.37(1)–0.52(3)	37(8)–51(6)	3.2(1)–7.5(1)× 10^−5^	5
B″	Very leaky Schottky	0.81(1)–1.94(1)	24(3)–73(15)	1.4(1)–15.6(1) × 10^−4^	50
C	Linear ohmic	0.06(2)–0.08(3)	32(2)–43(4)	0.7(6)–2(1) × 10^−6^	5
D	Linear ohmic	0.05(2)–0.09(4)	40(9)–49(6)	0.4(6)–1.8(1.6) × 10^−6^	5

^a^ The TLM variability is further shown in the supplementary material.

**FIG. 2.**
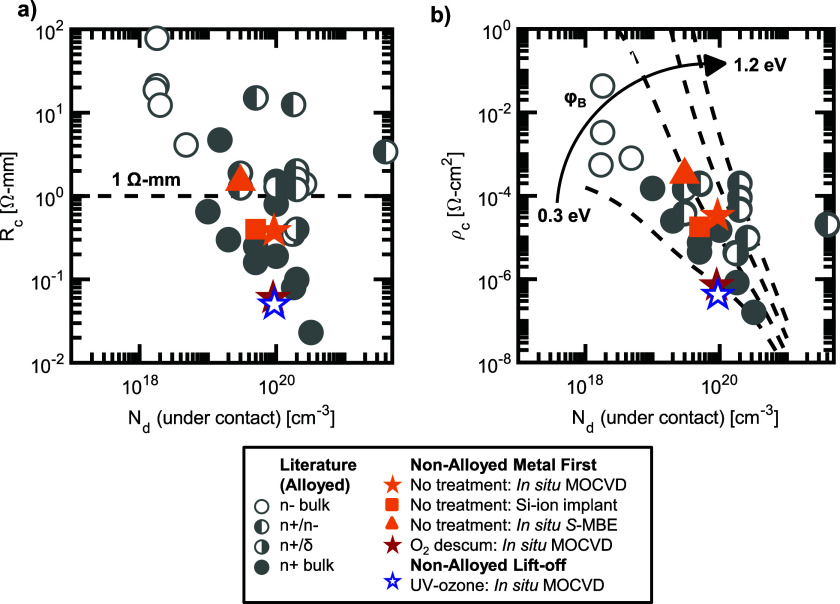
Benchmarking of (a) *R*_c_ and (b) *ρ*_c_ for the non-alloyed samples reported in this work is plotted in color against historically reported contact resistance values on (010) *β*-Ga_2_O_3_ (shown in gray; tabulated in Table S1 of the supplementary material).^13,14,22,30–43^ In orange, the star, square, and triangle represent samples B, B′, and B″, respectively. The red and blue stars represent samples C and D, respectively. The contacts on samples C (red) and D (blue) are among the lowest resistance Ti/Au contacts to (010) *β*-Ga_2_O_3_ reported to date. The 0.05 Ω-mm contacts on sample D represent the lowest reported contact resistance to sub-10^20^ cm^−3^ doped Ga_2_O_3_ to date. The lowest contact resistance values that are measurable in this study are limited by the CTLM geometries; any contacts better than 0.05 Ω-mm or 5 × 10^−7^Ω-cm^2^ necessitate different TLM designs for improved accuracy.

For sample B, CTLM gives values for *R*_c_ of 0.37(4)–0.42(3) Ω-mm, which corresponds to a *ρ*_c_ of 1.2(2)–1.8(1) × 10^−5^ Ω-cm^2^. Sample B′ gives similar values for an *R*_c_ of 0.37(1)–0.52(3) Ω-mm, with a corresponding *ρ*_c_ of 3.2(1)–7.5(1) × 10^−5^ Ω-cm^2^, and sample B″ gives higher values for an *R*_c_ of 0.81(1)–1.94(1) Ω-mm, with a corresponding *ρ*_c_ of 1.4(1)–15.6(1) × 10^−4^ Ω-cm^2^. Again, the higher contact resistance for sample B″ can be attributed to the lower doping density. In addition, in sample B″, a decrease in *R*_c_ with applied bias shown in Fig. 1(c) highlights the leaky Schottky contact behavior noted previously. In comparison, sample B observes a near-constant *R*_c_ shown in Fig. 1(d), indicating linear contact behavior. The *R*_c_ behavior in sample B′ is similar to that of sample B and is included in Fig. S2 of the supplementary material. The full IV curves and TLM analysis for samples B–B″ are also included in the supplementary material. The non-alloyed contact resistances of samples B–B″ (plotted in orange) are benchmarked in Fig. 2 and are within the range of values reported for similarly doped samples. Contacts on samples B and B′ further show limited contact variability across the sample with 1*σ* variation of ±0.03 Ω-mm for sample B and ±0.06 Ω-mm for sample B′, while sample B″ has higher variability due to the leaky Schottky nature of the contacts. Wafer maps can be found in the supplementary material.

The addition of the active oxygen treatment prior to metal-first contact deposition for sample C results in a lower *R*_c_ of 0.06(2)–0.08(3) Ω-mm, which corresponds to a *ρ*_c_ of 0.7(6)–2(1) × 10^−6^ Ω-cm^2^, representing ∼2 orders of magnitude improvement in *ρ*_c_. Sample C also exhibits a constant *R*_c_ with applied bias, as shown in Fig. 1(e), indicating linear contact behavior. Similarly, while the contacts fabricated on a lift-off-processed surface for sample A with a short UV-ozone treatment are not-conductive, the lifted-off contacts on sample D with an hour-long UV-ozone treatment have a low *R*_c_ of 0.05(2)–0.09(4) Ω-mm, which corresponds to a *ρ*_c_ of 0.4(6)–1.8(1.6) × 10^−6^ Ω-cm^2^. Note, the uncertainty in *ρ*_c_ originates from a compounding of the uncertainty in R_c_ and R_sh_ and clearly demonstrates the limitations of these TLM patterns at measuring small contact resistances. The *R*_c_ behavior in sample D is similar to that in sample C; the IV curves and TLM analysis for samples C and D can be found in the supplementary material.

Sample C also shows very low contact variability across the sample (<0.01 Ω-mm; see the wafer map in Fig. S4). Sample D has higher variability (0.02 Ω-mm, Fig. S5), which is still more uniform than samples B–B″. This suggests that oxygen descum or UV-ozone treatment improves contact uniformity; however, many of the samples used in this study have very small area (<1 cm^2^) and therefore contain a relatively small number of TLM patterns located near the edges of the sample. Uniformity improvements can be better confirmed by studies on larger area samples. The contacts on sample C and D represent the lowest reported contact resistances to sub-10^20^ cm^−3^ doped *β*-Ga_2_O_3_, as well as the second-lowest reported contact resistance to (010) *β*-Ga_2_O_3_ to date (well below the oft-quoted 1 Ω-mm criteria for practical ohmic contacts). These contacts are benchmarked (sample C in red and sample D in blue) against historically reported ohmic contacts (gray) in Fig. 2. The lowest contact resistance values that are measurable in this study are limited by the CTLM geometries; any contacts better than 0.05 Ω-mm or 5 × 10^−7^ Ω-cm^2^ necessitate different TLM designs for improved accuracy.

Our prior report demonstrated that metal-first processing provided improved contact consistency and repeatability over conventional lift-off processing, possibly attributable to contamination of the interface by organics from the photolithography process.^20^ However, this work indicates that even metal-first processing on as-grown material exposed to ambient air can be improved by adding a short active oxygen treatment on the as-grown surface prior to contact-metal deposition. Furthermore, long UV-ozone treatments appear to have the same effect for lift-off processed contacts. Both active oxygen and UV-ozone are particularly effective treatments for removing organic contamination, indicating that even nominally metal-first contacts on as-grown materials that do not receive one of these treatments may inadvertently incorporate very thin (sub-nanometer) layers of organic contamination. To understand both the source of the interface contamination and poor contact quality on lifted off contacts, as well as the improvement of even metal-first interfaces with the addition of oxygen treatments, further interface characterization is required. STEM analysis of samples A–D examined their microstructure and chemical composition. With high spatial resolution, STEM directly probes the local lattice and the electronic structures to determine whether a barrier at the interface separates linear ohmic and non-conductive contacts.^44,45^

**FIG. 3.**
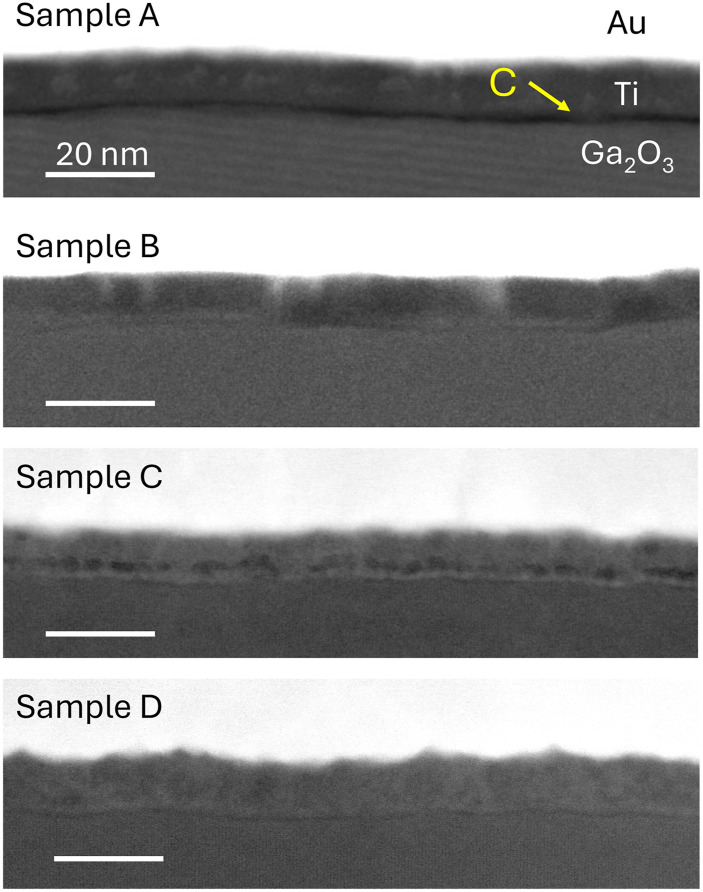
Overview of ADF-STEM cross-sectional images of the metal contact Au/Ti/Ga_2_O_3_ interface. Sample A (non-conductive) shows an ∼1 nm thick contamination layer between the Ti and Ga_2_O_3_ interface, which is not observed in linear ohmic contact samples B–D. The positive identification of carbon as the contamination layer is presented in forthcoming EELS data in Fig. 5.

Figure 3 captures wide field-of-view ADF-STEM images of the Au/Ti/Ga_2_O_3_ interface. The bright saturated contrast corresponds to Au, the element with the highest atomic number. In comparison, dark contrast highlights regions of low atomic number (Z) or low density. In sample A, an ∼1 nm dark contrast layer is apparent between the Ti and Ga_2_O_3_ layers, indicating low Z element contamination. This discrete layer is absent in samples B–D, suggesting that a barrier layer at the Ti/Ga_2_O_3_ interface causes non-conductive devices. Sample C does show clusters of dark contrast within the Ti layer, although not at the Ti/Ga_2_O_3_ interface. These clusters are hypothesized to be TiO_*x*_ incorporation owing to non-ideal deposition of Ti, stemming from suboptimal heating of the Ti source prior to deposition. In comparison, samples B–D show increased contrast right above the Ti/Ga_2_O_3_ interface, signaling a higher Z element incorporation. Previous studies have shown that Ti oxidizes and reduces Ga_2_O_3_ to form TiO_*x*_ and defective Ga_2_O_3_ by room temperature diffusion. We also note that sample B exhibits no contrast variations within the Ga_2_O_3_ layer, indicating a fully recovered lattice structure near the ohmic contact after Si implantation.

**FIG. 4.**
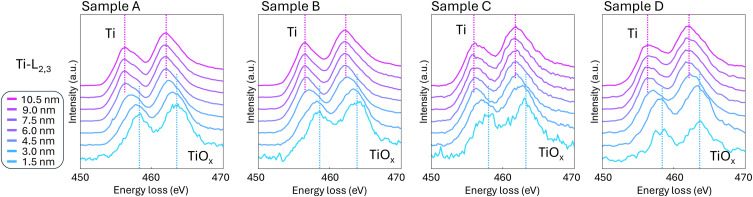
Normalized EELS spectra of Ti-L_2,3_ edges in samples A–D from the Ga_2_O_3_ interface. The spectra reveal a progression in the Ti valence state, transitioning from non-crystalline TiO_*x*_ near the Ga_2_O_3_ interface to metallic Ti further away. The magenta and blue dashed lines mark the peak positions of Ti and TiO_*x*_, respectively, illustrating the change in Ti bond state. Samples A–D all show Ti gettering oxygen from Ga_2_O_3_ to form TiO_*x*_ regardless of interface carbon.

The chemical nature of the Ti contact was explored using EELS to provide information on its electronic state. The spectra in Fig. 4 represent a line profile of summed 1.5 nm regions to produce the high signal-to-noise series. The maximum peak of the Ti-L_2,3_ edges was normalized. The increase in noise of the spectra toward the Ga_2_O_3_ interface is a product of less Ti signal due to the amorphous nature and less Ti presence near the rough Ga_2_O_3_ interface. Both the Ti-L_2_ and Ti-L_3_ edges show a gradual shift to higher energies in the lower ∼6 nm near the Ga_2_O_3_ interface, indicating an increase in the Ti charge state.^8,46–48^ The shift confirms that Ti scavenges oxygen from Ga_2_O_3_ to form amorphous TiO_*x*_ in all samples analyzed by STEM, as previously reported by XPS.^20,21^

**FIG. 5.**
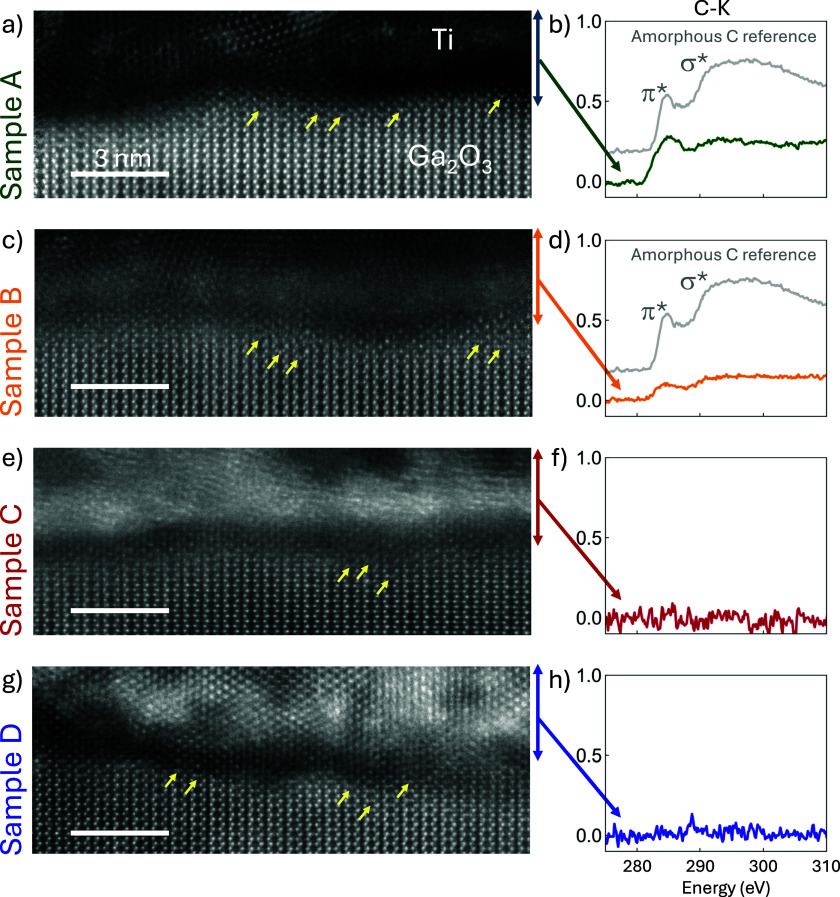
(a), (c), (e), and (g) Atomic resolution ADF-STEM images of the Ti/Ga_2_O_3_ interface along the [001] zone axis in samples A–D. All samples show Ga interstitial columns indicated by yellow arrows near the Ga_2_O_3_ surface. A dark contamination layer separates Ti from Ga_2_O_3_ in sample A, while the transition from Ga_2_O_3_ to Ti results in near-perfect adherence in samples B–D. The photoresist residue therefore creates an insulating barrier not just by its presence but also by distancing the bonds between Ti and Ga_2_O_3_. (b), (d), (f), and (h) Normalized C–K EELS spectra acquired from the region across the Ti and Ga_2_O_3_ layers between the double-headed arrows of in (a), (c), (e), and (g), respectively. Samples A and B show presence of bonded carbon, plotted against an off-set amorphous carbon reference in gray. Samples A and B show the signal of carbon contamination, while samples C and D do not show any carbon signature. The C–K EELS spectra shape in samples A and B are distinct from the amorphous carbon and each other, signaling different sources of contamination. The carbon signature decreases from samples A–D, correlating with the measured ohmic contact resistance.

Atomic resolution ADF-STEM images and EELS spectra in Fig. 5 reveal further details of the contamination layer and microstructure. The Ga_2_O_3_ surfaces of all samples (A–D) are not atomically flat, showing local height variations. In sample A, an ∼1 nm dark contamination layer separates Ti from the Ga_2_O_3_ surface [Fig. 5(a)]. The yellow arrows indicate Ga interstitial columns present in all samples. Hints of *γ*-Ga_2_O_3_ near the Ga_2_O_3_ surface are also present and can be identified as the larger hexagonal pattern with an atom sitting in the middle of the hexagon.^7,49^ These structural defects align with previous reports of the *γ*-phase commonly observed with Ga interstitials at the Ga_2_O_3_ surfaces.^49,50^ The *γ*-phase, with lower density and high Ga vacancy accommodation, likely results from oxygen loss to TiO_*x*_ formation, as discussed previously. In comparison with sample A, samples B–D show near-perfect adherence between the TiO_*x*_ and the disordered Ga_2_O_3_ surface [Figs. 5(c), 5(e), and 5(g)]. While *γ*-Ga_2_O_3_ has been theorized to contribute to the non-ideal ohmic contact region,^49^ its presence does not correlate with non-conductive IV behavior in these samples.

EELS spectra across the Ti–Ga_2_O_3_ interface identify carbon as the primary factor that affects high contact resistance. Each spectrum in Fig. 5 represents a summed region spanning the Ga_2_O_3_ interface to 3 nm in the Ti layer indicated by the double arrows. The C–K EELS spectra are normalized to the area under the Ti-L_2,3_ edge to account for lamella thickness variation, allowing for quantitative comparison. Samples A and B detect carbon at the Ti–Ga_2_O_3_ interface, as shown in Figs. 5(b) and 5(d). The measured C–K EELS edges in sample A are distinguishable from the amorphous carbon referenced by the gray curve by the depression of the C–K σ* peak, indicating that the carbon incorporated within the sample has a different bonded character. This bonded carbon is likely a residual photoresist, despite the multiple cleaning steps outlined in Sec. II. Sample B also shows a carbon signature, but at a lower intensity and distinct from sample A by the depressed C–K π* peak. We attribute the carbon signature in sample B to adventitious carbon found on the surface of air exposed samples. In comparison, samples C and D exhibit no carbon signatures.

Notably, previous studies using depth-resolved x-ray photoelectron spectroscopy were unable to detect the C contamination layer observed here by EELS in sample A.^20^ This can be attributed to the rough interface noted above; XPS is a highly surface sensitive technique; therefore, any roughening of the interface would effectively dilute the signal of a single monolayer of C such that it is below the 0.1%–1% detection limit of XPS. Spatially non-uniform sputtering during depth-resolved measurements caused by sample charging, especially in the case of patterned metallic features resulting in a lateral variation in sample conductivity, can further exacerbate this effect.

It should also be noted that excessive exposure to active oxygen species may decrease Si doping efficiency due to the formation of compensatory defects such as Ga vacancies according to both first-principles calculations and experimental observations of the activation of implanted Si in *β*-Ga_2_O_3_.^7,51^ Our results in this work demonstrate that both 1-h long UV ozone and 5-min 100 W O_2_ descum are effective to remove sub-monolayer of surface carbon while retaining the Si doping efficiency near the Ga_2_O_3_ surface.

The level of carbon contamination measured by EELS correlates directly with the measured ohmic contact resistance. Similar to known Si contamination on the Ga_2_O_3_ surface,^24^ the carbon signature in sample B, which was never in contact with photoresist, could be attributed to organic contamination on the surface from air exposure between ion implantation/activation annealing and ohmic contact processing. The samples were grown in batches and may have spent significant amounts of time in ambient air or nitrogen box environments prior to device processing. The correlation of carbon contamination with ohmic contact resistance highlights the importance of proper surface preparation and cleaning and indicates that for best practice even for metal-first contact processes should include an organic-removing treatment such as UV-ozone or active oxygen plasma prior to contact metal deposition to minimize ambient air contamination.

The presence of air contamination on Ga_2_O_3_ surface can also be measured by ESR spectroscopy. Although adsorbed impurities, such as siloxanes^24^ or carbon, on the surface of *β*-Ga_2_O_3_ are unlikely to host paramagnetic point defect centers of definite charge and spin states due to their amorphous nature, they can nevertheless host local magnetic moments that form two-level systems (TLSs) with a broad energy landscape.^52^ This is especially likely since both silicon and carbon have stable isotopes that possess a nuclear spin.

**FIG. 6.**
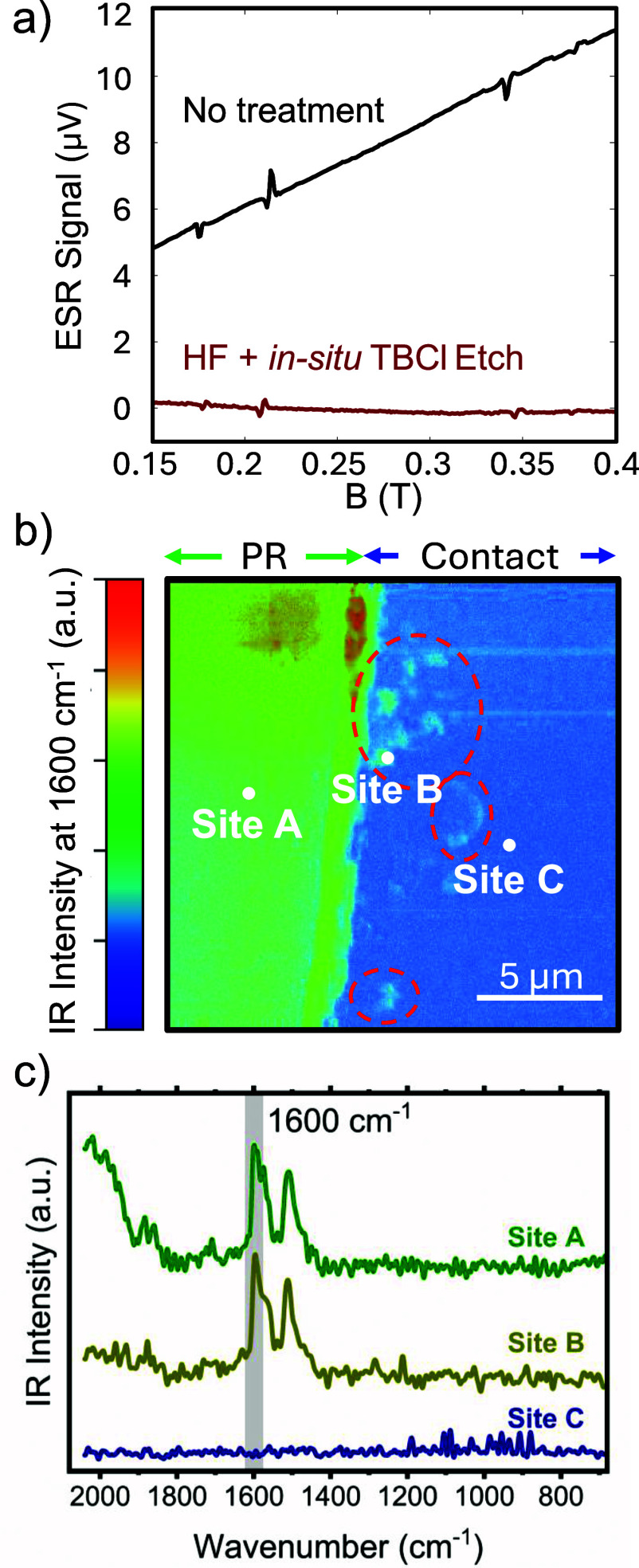
(a) ESR spectra of *β*-Ga_2_O_3_ films on Fe-doped (010) *β*-Ga_2_O_3_ substrates without surface treatment and with a 30-min HF dip and *in situ* TBCl etching before MOCVD growth (samples E and F). The broad spin background is attributed to adsorbed impurities that can be eliminated by cleaning the substrate surface. The sharp ESR signals correspond to spin signatures with well-defined atomic environments. (b) AFM-IR 2D photothermal IR absorption map of lift-off sample G before Ti/Au electron-beam evaporation with a 2 min active oxygen descum. The region of photoresist (PR) and the nominally clean region intended for contacts are labeled between the double arrows. IR intensity indicates the presence of aromatic ring vibrations expected in photoresist. Regions of carbon contamination in the contact region are circled by the red dashed lines. (c) IR point spectra from the locations indicated by the white dots in (b). The gray shaded region in the presented spectra indicates the approximate wavenumber region (center ± FWHM) used to acquire the spectral maps in (b). Site B acquired in the contact region shows a photothermal response indicative of carbon contamination similar to the IR spectra acquired from the site A acquired on photoresist. Site C, on the other hand, does not show any peaks at 1510 or 1600 cm^−1^, indicating a photoresist-free Ga_2_O_3_ surface.

Figure 6(a) shows the ESR spectra of UID MOCVD *β*-Ga_2_O_3_ films grown on Fe-doped (010) *β*-Ga_2_O_3_ substrates collected at 9.936 GHz with the magnetic field along the [010] axis (samples E and F). Sharp signatures of Fe^3+^ at the octahedral Ga(II) site can be seen as peaks in the spectra of both samples E and F at 0.213 and 0.342 T^53–55^ with additional ESR signatures of unknown origin at 0.176 and 0.379 T. In addition to these sharp ESR lines, a broad spin-background is seen in sample E. This broad background is completely eliminated in sample F that treated the substrate with HF and an *in situ* TBCl etch. We attribute this spin-background signal to magnetic moments hosted by amorphous adsorbates on the surface of the Ga_2_O_3_ substrate. From our previous studies,^24,26^ high levels of impurities including C and Si are found at the regrown interface in samples that are cleaned in solvent only prior to growth, while these impurity levels are reduced by orders of magnitude with HF and TBCl treatments. ESR spectroscopy is therefore capable of measuring magnetic moments stemming from amorphous adsorbates separately from paramagnetic point defects in the *β*-Ga_2_O_3_ lattice, the latter of which are not removed by surface treatments. The spin background seen in the ESR spectra, in agreement with STEM-EELS measurements on metal-first sample B, thus indicates the presence of amorphous adsorbates on untreated Ga_2_O_3_ substrates.

Photoresist contamination on the Ga_2_O_3_ surface can also be detected via a non-destructive method using AFM-IR. Sample G was subjected to a 2-min 100 W active oxygen descum after the sample had undergone the pattern development lift-off process. An AFM-IR map was acquired over patterned regions of photoresist and nominally clean areas before Ti/Au contact deposition. The IR intensity at 1600 cm^−1^, chosen to highlight regions of carbon contamination, is presented as a 2D map in Fig. 6(b). Patches of carbon residue are circled by the red dashed lines.

IR spectra at selected points on the 2D map are shown in Fig. 6(c). The IR spectra obtained from site A is a representative of the IR spectra throughout the from the photoresist-covered regions, which shows prominent peaks at 1510 and 1600 cm^−1^, likely indicative of phenyl and carboxyl groups, respectively. Site B provides an IR spectrum representation of those captured from nominally clean areas after patterning that show a photothermal response at 1510 and 1600 cm^−1^ that arise from the aromatic ring vibrations of the cresol novolak-based photoresist (greenish-blue aqua color).^56^ The IR point spectrum from site B is similar to the IR spectrum obtained from site A, confirming the presence of residual photoresist. In comparison, site C represents nominally clean areas with no evidence of photoresist. The IR spectra obtained at site C do not show the characteristic photoresist peaks at 1510 or 1600 cm^−1^. Therefore, sample G shows that the lift-off patterning process leaves patches of photoresist behind on the Ga_2_O_3_ surface, and a two-minute 100 W active oxygen descum is not sufficient to eliminate carbon contamination before metal contact deposition, consistent with STEM finding on sample A. Incomplete photoresist removal can further become baked onto the sample during ICP-RIE etching. Consequently, sample D that underwent an hour long UV ozone cleaning is the best performing lift-off ohmic contact when the photoresist residue is completely removed. These results demonstrate the ability of AFM-IR to serve as a non-destructive characterization technique that can be implemented as a process check for confirming clean contact interfaces and/or detecting nanoscale photoresist residue on fabricated samples.

The AFM-IR measurements were further corroborated by TOF-SIMS measurement of sample G in the photoresist patterned and open areas. Measurement of the photoresist (near site A in Fig. 6) identified several C_x_H_y_N^+^ fragment peaks that are characteristic of the photoresist. Signatures of these peaks are detected in the supposedly open area (near site B in Fig. 6), indicating that photoresist residue is present even with the 2 min oxygen descum. Ga^+^ signal is also detected in the open area, indicating that the photoresist residue is likely less than a monolayer thick and/or only partial coverage of the sample surface. The mass spectra are included in the supplementary material.

## IV. CONCLUSION

Overall, the variation in Ti/Au ohmic contact quality as a function of lift-off and metal-first processing has been investigated. STEM-EELS measurements find that the level of carbon contamination at the Ga_2_O_3_/Ti interface correlates directly with the measured ohmic contact resistance. We find non-conductive contacts fabricated by a lift-off process to contain significant photoresist carbon residue and hypothesize that the large variations in contact performance previously reported result from similar remnant photoresist. We further report that an hour-long UV-ozone treatment is shown to remove the photoresist residue to attain linear ohmic contacts with a contact resistance R_c_ of 0.05 Ω-mm. We propose that AFM-IR can be used as a non-destructive scanning probe measurement for mid-process diagnostic to ensure surface cleanliness prior to contact deposition for critical device processes. On the other hand, metal-first processing shows linear ohmic non-alloyed contact behavior on an as-grown sample. However, the contact interface still contains a carbon EELS signature from organic contamination due to air exposure that can also be detected by ESR spectroscopy. Addition of a 5 min active oxygen descum on the as-grown surface prior to contact metal deposition eliminates this adventitious carbon and results in a ∼5× reduction in contact resistance to 0.06 Ω-mm, the second lowest reported contact resistance to (010) *β*-Ga_2_O_3_ to date. We conclude that non-alloyed contacts are attainable via both lift-off and metal-first contact processing when surface carbon is managed appropriately.

## SUPPLEMENTARY MATERIAL

The supplementary material contains a detailed explanation of the MOCVD growth, ion implantation, and complete fabrication process of sample A. Additional data of the IV curves and complete TLM analysis of samples B, B′, B″, C, and D are included, along with the wafer map. Finally, a table of historically reported Ti/Au contact resistance values plotted in gray in Fig. 2 and ToF-SIMS data of sample E are included.

## Data Availability

The data that support the findings of this study are available within the article and its supplementary material.
